# The Role of the Intestinal Epithelium in the “Weep and Sweep” Response during Gastro—Intestinal Helminth Infections

**DOI:** 10.3390/ani12020175

**Published:** 2022-01-12

**Authors:** Piotr Bąska, Luke James Norbury

**Affiliations:** 1 Division of Pharmacology and Toxicology, Department of Preclinical Sciences, Institute of Veterinary Medicine, Warsaw University of Life Sciences, 02-786 Warsaw, Poland; 2 School of Science, STEM College, RMIT University, Bundoora 3083, Australia; luke.norbury2@rmit.edu.au

**Keywords:** helminths, immune response, IEC, immunomodulation, gut immune response, immune regulation

## Abstract

**Simple Summary:**

The immune system actively combats intruders such as bacteria, viruses, fungi, and protozoan and metazoan parasites using leukocytes. During an infection white blood cells are activated to internalize bacteria or viruses and release a number of molecules to kill pathogens. Unfortunately, those mechanisms are ineffective against larger intruders like helminths, which are too large to be killed by a single immune cell. To eliminate gastro-intestinal helminths an integrated response involving the nervous, endocrine, and immune systems are used to expel the parasites. This is achieved through increased gut hydration and muscle contractions which detach worms from the gut and lead to release outside the body in a “weep and sweep” response. Epithelial cells of the intestine are significant players in this process, being responsible for detecting the presence of helminths in the gut and participating in the regulation of parasite expulsion. This paper describes the role of the gut epithelium in detecting and eliminating helminths from the intestine.

**Abstract:**

Helminths are metazoan parasites infecting around 1.5 billion people all over the world. During coevolution with hosts, worms have developed numerous ways to trick and evade the host immune response, and because of their size, they cannot be internalized and killed by immune cells in the same way as bacteria or viruses. During infection, a substantial Th_2_ component to the immune response is evoked which helps restrain Th_1_-mediated tissue damage. Although an enhanced Th_2_ response is often not enough to kill the parasite and terminate an infection in itself, when tightly coordinated with the nervous, endocrine, and motor systems it can dislodge parasites from tissues and expel them from the gut. A significant role in this “weep and seep” response is attributed to intestinal epithelial cells (IEC). This review highlights the role of various IEC lineages (enterocytes, tuft cells, Paneth cells, microfold cells, goblet cells, and intestine stem cells) during the course of helminth infections and summarizes their roles in regulating gut architecture and permeability, and muscle contractions and interactions with the immune and nervous system.

## 1. Introduction

“Weep and sweep”—this term is used to describe the host response against gastro-intestinal (GI) helminths. The main features of this phenomenon are an increase in fluid in the gut lumen and enhanced smooth muscle contraction; this facilitates expulsion, rather than the killing of the intruder [1]. Although the premise of “weep and sweep” is simple, the mechanisms that regulate this phenomenon are complicated and require the interplay of the immune, endocrine, and nervous systems, not only through chemical mediators but through physical interactions. The response needs to be strong enough to efficiently terminate infection but gentle enough to only cause minor or at least acceptable injuries. A balance between the three classical Th_1_, Th_2_, and Th_reg_ responses needs to be maintained to avoid any dysregulation which may lead to immune-mediated injuries.

**Figure 1:**
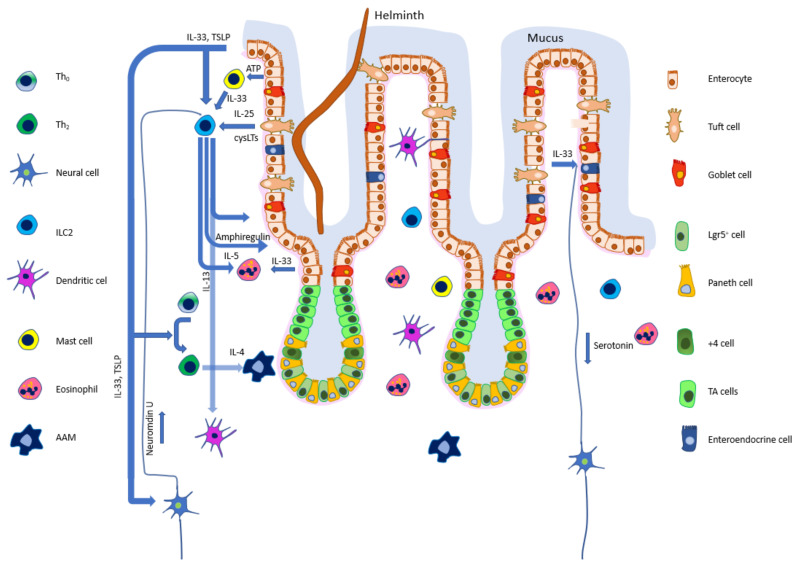
Helminth induced interactions between epithelial, neural, and immune cells. Upon infection tuft cells (TC) release IL-25 and other intestinal epithelial cells (IEC) secrete TSLP, IL-33, and ATP, signaling tissue damage. Group 2 innate lymphoid cells (ILC2) stimulated by alarmins release IL-4, IL-5, IL-9, and IL-13, inducing goblet cell secretion, alternatively activated macrophages (AAM), and eosinophilia. ILC2 also cooperate with the nervous system, and upon being stimulated by neuromedin U release amphiregulin which has tissue-protective properties. Enteroendocrine cells in response to IL-33 release serotonin which induces muscle contraction or epithelium permeability.

For the response to be efficient and balanced a number of regulatory mechanisms have evolved (schematically represented in Figure 1). The first stage in this complicated host-parasite interplay is host recognition of the parasite, which triggers several effector mechanisms. However, while it is easy to indicate the first step—recognition, our comprehension of the mechanisms underlying this process are still in their infancy. There are a number of fantastic reviews describing the mediators of immune responses [2], the systems (neural, immune, endocrine) involved [3,4,5], or scrutinizing known processes during helminth infections [6]. However, the purpose of this review is to sequentially describe the events beginning from helminth recognition through effector mechanisms. Unfortunately, it can be difficult to describe the chronology of events since the cooperation of numerous host organs and systems commences and occurs concurrently. Moreover, mediators and effector cells participate in various components and stages of the weep and sweep phenomenon. Nevertheless, it is hoped this review will bring attention to little-known facts of, and give a new insight into, the “weep end sweep” response.

## 2. Helminths

Helminths are an artificial grouping that is comprised of multicellular parasitic worms. They inhabit host tissues (GI tract, lungs, muscles, and other organs) and infections may be long lasting, even up to 20 years. Helminths are a problem of both human and veterinary medicine. According to the CDC (US centers for disease control and prevention) up to 1.12 billion, 795 million, and 740 million people suffer from Ascaris, whipworm, and hookworms, respectively [7]. Helminth infections may lead to cognitive impairment [8], tissue damage [9], pregnancy complications [10], and numerous other symptoms and can sometimes be fatal [11]. The high prevalence, detrimental effects, and increasing drug resistance of helminths [12,13] have led to much research on developing vaccines [14,15,16,17,18]; however, despite promising results, there is still much to be done to achieve final success. Fully eradicating helminth infections may even in some aspects be controversial, since they may mitigate autoimmune diseases [19,20,21] and allergies [22,23] by dampening Th_1_ and inducing Th_2_ host immune responses [24,25]. The resulting main effects of this response typically are the presence of alternatively activated macrophages, tissue repair, eosinophilia, and the production of IgE [26,27]. This state can be beneficial for both the host and the parasite since Th_2_/Th_reg_ responses protect tissue and induce wound healing in the host, while the parasite can complete its life cycle and achieve reproductive success. There are a variety of helminth species, each with its own host-parasite interplay which influences infection progression and outcome; moreover, host genetic background [28] and parasite strain [29,30] can also impact the immune response.

## 3. Structure of the Gut Epithelium

The intestine is a complex organ engaged in digestion that is also constantly in contact with bacteria and toxins. To fulfill its function the small intestinal epithelium is organized into villi and crypts, enlarging the surface engaged in nutrients absorption [31] (whereas the large intestine is composed of crypts only). All epithelial cells lining the intestine come from Lgr5+ intestinal stem cells (ISC), but in the case of tissue damage other cells may acquire stem-cell-like phenotypes through dedifferentiation [32]. Lgr5+ cells reside at the bottom of crypts [33] constantly renewing the epithelium by differentiating into enterocytes, microfold (M) cells, enteroendocrine cells (EEC), goblet cells, Paneth cells, and tuft cells (TC) lineages.

Enterocytes are the most common differentiated intestinal cell; they are polarized cells, the apical side of the cell membrane creating a brush border (composed of microvilli) enhancing the surface area for contact with gut lumen content. Enterocytes are accountable for epithelium integrity, adsorption of digested food, and regulation of water homeostasis [34]. Goblet cells are dispersed among enterocytes, they are mucin producers and may transfer antigens to the lamina propria. Another cell population engaged in interactions with immune cells are M cells. They have an invaginated basal cell membrane (called an “M cell pocket”) and reduced microvilli located on the apical surface compared to other epithelial intestinal cells. The modified microvilli allow for easier luminal antigen capture and the M cell pocket facilitates the passing of antigens to macrophages or dendritic cells [35]. Paneth cells occupy the basal compartment of crypts and release a battery of antimicrobial proteins. Enteroendocrine cells (EEC) are widespread throughout the gastrointestinal (GI) tract and may be subdivided into at least ten different populations [34]. EEC release hormones that regulate digestion, act as neuromediators and can contact the immune system with neuromotoric actions. The last population are TCs, these cells are enriched with receptors that recognize lumen content and regulate a response upon stimuli; they are a cell population substantially engaged in the immune response against helminths.

## 4. Epithelial Cells Modulate the Immune Response

The GI region is a highly specific place for immune system function. It has to be able to tolerate non-pathogenic, commensal microflora and food antigens, as well as counteract pathogenic microflora and metazoan invaders, such as helminths. The intestinal epithelium is the 2nd barrier (behind mucus) to interact with these distinct foreign organisms. It is also an immune active tissue interacting with myeloid cells, lymphoid cells, and the nervous system, orchestrating recognition of pathogens and finetuning the effector mechanisms of responses. Upon stimulation, the intestinal epithelium exhibits altered gene expression [36] and releases distinct cytokines [37] promoting a shift in the immune response towards Th_2_ [38] or Th_reg_ phenotypes [37,39]; this is in contrast to the lung epithelium where an inflammatory response is more desired [40].

Each helminth species is unique and may trigger distinct mechanisms since each occupies its own niche in the GI tract and has different biological characteristics (e.g., feeding, mating). Most data regarding the role of intestinal epithelial cells (IECs) during helminth infections comes from research with four nematodes: *Trichuris muris*, *Trichinella spiralis*, *Nippostrongylus brasiliensis*, and *Heligmosomoides polygyrus*. *T. muris* localizes in the caecum with its anterior part anchored in “syncytial tunnels” formed from modified epithelial cells [41], whereas *T. spiralis*, *N. brasiliensis* and *H. polygyrus* reside in the small intestine [42]. *H. polygyrus* attaches to the intestine through coiling around the villi on epithelial cells [43], *N. brasiliensis* feeds on host villus tissue [44] and *T. spiralis* occupies a few epithelial cells creating a multicellular niche [45]. Despite the different biology and living niches, their impact on the host immune response shows a number of common features (reviewed below). Studies over the last 15 years have focused on investigating the immune interplay between parasites and the host immune system (cytokine profiles, gene expression patterns), whereas other aspects like physical contact between the worm and host epithelium remained somewhat neglected. It is now clear that both parasite physical interactions with host tissues as well as the impact of molecules released by parasites on the host are part of an extremely complicated process and should be investigated in complement with molecular research. Upon infection GI parasites penetrate through the mucus barrier and provoke a host reaction; sometimes it is impossible to define whether a particular phenomenon is an outcome of helminth actions or the host immune response. Nevertheless, despite the very first interactions remaining elusive, initial events occur in response to IL-25, IL-33, and TSLP signaling pathways [46]. The hallmark of the anti-GI helminth response are goblet cell hyperplasia, eosinophilia, enhanced peristalsis, and release of Th_2_ type cytokines. However, an exacerbated host response may lead to detrimental effects, and negative feedback loops are initiated to fine-tune the response.

## 5. Mucus as the First Physical Frontier

Mucus acts as a physical, gel-like, barrier separating the epithelium from microbiota and gut contents, and it differs in structure and composition along the intestinal tract. It also acts as a lubricant and contains immunoactivite components. The simplest, mucus-dependent, mechanism assisting infection termination is the physical entrapment of worms, facilitating detachment from the tissues to the gut lumen and expulsion [42]. This occurs during *T. spiralis* and *N. brasiliensis* infection [42] but does not seem to be crucial to remove the parasites from the intestine, and more sophisticated mechanisms have evolved to combat helminth infections. Mucus has a very complicated structure; in the small intestine mucus is composed of one layer loosely attached to epithelial cells, shows relatively high permeability, and repels bacteria, whereas colon mucus may contain two layers with a more complex composition [47], possibly dependent on the fecal content [48]. The mucus structure is stabilized mainly due to Muc2, the most substantial mucus protein [49]. Dynamic changes in mucus composition during infections may have beneficial results. A change in mucus composition, e.g., the appearance of Muc5ac, seems to be crucial for the expulsion of various parasites, e.g., *T. muris*, *T. piralis*, and *N. brasiliensis* [50].

Other anthelmintic properties of mucus are associated with post-translational modifications, where enhanced mucus sulphation (in species *Tscherskia triton* and *Cricetulus griseus*) is associated with higher resistance to infection [51]. Sulphation of mucins is also beneficial during *T. muris* infection since it facilitates worm elimination, whereas another modification—sialylation—is associated with chronic infection [52]. The beneficial mechanism of sulphation is probably based on decreased vulnerability to parasite proteases [42].

Being continually exposed to bacteria and parasites, mucus produced by the gut epithelium needs to be constantly renewed and recompositioned. This process stays under strict immune control (reviewed elsewhere [53,54]), however, regulation by Trefoil Factor Family proteins (TFFs) is worth mentioning due to the direct cooperation with another regulator of the immune response—amphiregulin—which is released in response to IL-33 by group 2 innate lymphoid cells (ILC2s) and protects tissue from damage [55] and facilitates worm expulsion [56]. Firstly, TFF3 enhances rheological mucus properties, improving barrier stability. This action is likely mediated by binding Leucine-rich repeat and immunoglobulin-like domain-containing nogo receptor-interacting protein 2 (LINGO2) which prevents Epidermal Growth Factor Receptor (EGFR) signaling. LINGO2 is present not only in IEC but also in hematopoietic cells [57] so its role needs further exploration. However, the molecular mechanism of TFF3 and LINGO2 has been characterized. TFF3 binds to LINGO2 and abrogates its blocking effect on EGFR signaling [57] allowing for amphiregulin binding and inducing tissue-protective mechanisms [55] through activation of antiapoptotic STAT3 [58].

## 6. Epithelium as the Second Physical Frontier

The intestinal epithelium is a firm physical barrier between the organism and its gut lumen. The stability of the IEC is associated with the development of tight junctions (TJs) between cells, composed mainly of occludin, junctional adhesion molecule (JAMs), tricellulin, and claudins [59] with the latter considered key players in regulating gut permeability [60]. TJ proteins are anchored inside cells through interactions with actin [61], stabilizing the whole structure of the epithelium. This enacts a compromise in function for the intestinal epithelium between acting as a physical barrier and permeability for communication.

Helminth infections focally damage the intestinal epithelium, the destruction increasing epithelium permeability. However, the host also actively increases intestinal epithelium permeability when mounting an immune response to facilitate the transport of complement, antibodies, and immune cells into the gut lumen [62]. This enhanced permeability during infection is Th_2_ dependent as mice deprived of STAT6 (transcription factor—marker of Th_2_ response) fail to increase epithelium permeability [63]. The detailed mechanisms of increased permeability during infection are complicated and still to be resolved, nevertheless, there are data shedding light on this phenomenon, which appears to be substantially regulated by acetylcholine (ACh), histamine, serotonin, prostaglandin E2 (PGE2), and proteases. ACh is a neuromodulator with pleiotropic functions that binds and activates nicotinic and muscarinic receptors. MR3 (type 3 muscarinic receptor) deprived mice do not show an increase in epithelial permeability during infection [64] and may have an exacerbated Th_1_ response [64] which is detrimental during helminth infections. Histamine, serotonin, and PGE2, released by mast cells, regulate chloride ion secretion [65], while the proteases (also released by mast cells) degrade occludin, destabilizing TJs, facilitating *T. spiralis* expulsion [65].

## 7. Helminth Recognition

Mammals use pathogen-associated molecular patterns (PAMPs) to adjust immune responses to a particular foe, and are equipped with Toll-like receptors (TLRs) [66] or NOD-like receptors (NLRs) [67] that specifically recognize bacterial and virus antigens; however, the pattern recognition receptors (PPRs) for helminth PAMPs have not yet been fully defined. The classic pathway of antigen recognition and presentation involves the sampling of the gut lumen by dendritic cells (DC) followed by internalization and presentation of antigens via MHCII to T cells. However, there is also a surprisingly significant role for MHCII expressed on IECs in the gut, which seems to be attributed to ISC Lgr5+ cells which act as nonconventional antigen-presenting cells and orchestrate epithelium structure both during homeostasis and infections [68]. These cells are sensitive to cytokines and are promoted towards renewal, or differentiation to Paneth cells or TCs upon exposure to Th_reg_, Th_1_, and Th_2_ (and their cytokines), respectively [69]. MHCII ablation in ISC during *H. polygyrus* infection leads to impaired TC expansion, and MHCII ablation in the gut decreases worm expulsion [69].

MHC II is also expressed on M cells which use the complex to capture luminal antigens [70] and pass it to gut-associated lymphoid tissue (GALT). Despite interest in MHCII use by M cells to stimulate the immune response, our understanding of this process is still limited [71]. M cells appear to constantly probe for luminal antigens, which are then presented to immune cells. M cells are located in FAE (follicle associated tissue) [72] which contains a decreased number of Paneth and goblet cells, therefore the mucus layer is thinner and the release of antimicrobial peptides is decreased [73]. M cells play an important role in antibacterial immunity, associated with Th_1_/Th_17_, but their efficiency at clearing metazoan parasitic infections is still to be determined.

More effective sentinels during helminth infections are Dclk1 + TCs [4]. TCs [74] orchestrate cell homeostasis at the intestinal mucosal barrier through a positive loop via the constant release of IL-25, enabling low-level synthesis of IL-13 which promotes their differentiation through binding to IL-4Rα [75]. TCs proliferate upon infection by *N. brasiliensis* [75], *T. spiralis* [76], and *H. polygyrus* [77], with proliferation dependent on the Pou2f3 transcription factor [78]. TCs use chemosensing through taste receptors; TAS1R and TAS2R possess the ability to sense sweet, sour, and umami. However, recent findings show that evolution has adapted TCs to also identify metazoan invaders in the GI tract [79]. Human TAS1R and TAS2R are classes of G-protein-coupled receptors (GPCRs) [80] and transduce signals through interaction with G protein—gustducin [81]. It has been shown that mouse Tas2rs mediates the release of IL-5 upon stimulation with *T. spirals* or its secretory antigens; moreover, the infection changes the mRNA expression profile of Tas2r family members [76] which may imply distinct roles for particular Tas2r in *T. spiralis* recognition. The function of other Tas receptors has also been explored, including during *H. polygyrus* infection, results indicating that Tas1r3 is engaged in maintaining intestinal cell homeostasis during health, rather than being involved in directly mounting a response against nematode infections [82].

Further exploration of the role of taste receptors in helminth recognition should be undertaken in the context of microbiota colonizing the gut, especially those that release succinate. Recently Nadjsombati et al. [83] showed that *N. brasiliensis* also releases succinate and that dietary supplementation with this chemical decreases the worm burden via the mounting of a Th_2_ response. Moreover, GI helminth infections can affect the composition of gut microbiota [62], and it is possible that these changes can facilitate the recognition of helminths invaders due to impacts on different receptors while physical injury to the epithelium caused by the worms may also result in stronger signals and impacts on immune cells.

Other receptors possibly engaged in helminth recognition are TLRs. *T. spiralis* directly interact with TLR2 and TLR4 on DCs [84], and TLRs are also present on IEC [85]. However, recognition of *T. spiralis* ES by TLR2 and TLR4 skew DC toward a Th_reg_ response [84] and it is likely to be an intentional immunomodulatory action of parasites [86]. Alternatively, *T. muris* ES may directly activate enteroendocrine cells, in a TLR2-dependent manner [87], which would support a role for TLR2 in helminth recognition. Nevertheless, the particular epitope (amino acid sequence or sugar moiety) recognized by the TLR remains unknown and it is also yet to be determined if TLRs play a crucial role or are merely auxiliary receptors in helminth recognition. Summing up, the proven ability of TLRs to bind parasite antigens indicates a role in recognizing parasitic infections and justifies further investigation of this phenomenon.

## 8. The First Interplay between the Epithelium and Immune Cells

Upon entering the host gut, helminths through inducing physical injuries and as yet undefined signals induce the release of alarmins (IL-33, IL-25, and TSLP) by IECs. TCs are the main source of IL-25, whereas IL-33 and TSLP are released by other IECs [88]. All three act as mediators to promote ILC2s to release IL-13, which is crucial to the development of a Th_2_ response. Nevertheless, this process is severely impaired without cysteinyl leukotrienes (CysLT) also released by TCs in response to infection [89]. To produce and release IL-13, ILC2s need strong stimulation and activation of three main transcription factors, NF-kB, AP-1, and NFAT. The first two are activated by IL-25 [89] and IL-33 [90], while the last is activated through CysLTs [89]. This is probably an evolutionary process allowing prevention of Th_2_ response exacerbation in the first events upon contact. Neurons are the other population besides IEC and DC to have the ability to respond directly to parasites. Cardoso et al. showed that neurons release neuromedin U upon stimulation with *N. brasiliensis* [91], which due to the small distance between neurons and ILC2s—4.716 µm—activates the latter to orchestrate a Th_2_ immune response. The intracellular events that occur upon neuromedin U being bound by NmuR1 on ILC2s are similar to the ones induced by CysLTs, with activation of a Ca^2+^/calcineurin/NFAT pathway [91]. This data suggests the coordination of several pathways to induce an appropriate response. However, both the receptor on neural cells that binds to *N. brasiliensis* ES and the particular parasite antigen bound by the receptor remains elusive. To maintain an appropriate balance and regulate the response, the nervous system can also stimulate ILC2 cells by releasing norepinephrine which binds to the β_2_-adrenergic receptor (β_2_AR). This leads to decreased ILC2 proliferation and probably fine-tunes the Th_2_ response preventing chronic inflammation [92]. Neural regulation of ILC2 by NmuR1 and β_2_AR is likely to play the role of fast regulator, mediating effects before stimulating immune effector cells. The response begins, as mentioned, with ILC2 activation by IL-25 and neuromedin U which promotes ILC2 expansion, activation, and release of typical Th_2_ mediators: IL-5, IL-13, and to a lesser extent IL-4 and IL-9 [93]. This leads to eosinophilia, regulation of DC migration, development of alternatively activated macrophages (AAM), and cross-talk with Th_2_ cells [94].

## 9. Physical Expulsion of Parasites

Expulsion of GI nematodes is orchestrated by a Th_2_ response with main roles for IL-4 and IL-13. To eliminate large invaders, as nematodes are compared to bacteria, the balanced cooperation of various cells (epithelial, immune, and neural cells) needs to be synchronized. Hosts evoke a number of actions to dislodge parasites from their niche and remove them along with gut contents, including using an ‘epithelial escalator’, and increased gut motility and water content in the gut lumen. The epithelial escalator is a phenomenon based on increased epithelial turnover [41] which physically displaces worms from their habitat in the epithelial layer to the gut lumen, facilitating expulsion [95]. This event is synchronized with enhanced peristalsis and gut hydration associated with acetylcholine and serotonin release. ACh is a well-described neuromediator in the cholinergic neural system; however, it is also released by TC [96] and may act locally as an autocrine and paracrine hormone [97]. ACh is responsible for the regulation of epithelial cell proliferation and differentiation [98,99] and mucus secretion by goblet cells [100]. ACh‘s impact on intestine permeability is regulated by serotonin which is released by enterochromaffin cells (EC) (one of the enteroendocrine cell subtypes) in response to mechanical stimuli [101], IL-33 [102] (which is abundantly present during parasitic infections) or directly in response to parasitic antigens [102]. Serotonin stimulates cholinergic neurons via a neural pathway to release ACh which enhances ion and water transport to the gut lumen [101]. Nevertheless, this process during an infection is immune-dependent as mice deprived of Th_2_ cells fail to increase epithelium permeability during infection [103]. This constitutes the "weep" part of the response. The next component, "sweep", is associated with muscle contractions and is regulated by the cooperation of a hormone-immune circuit. The above-mentioned serotonin release by EC in response to IL-33 not only results in increased permeability but also enhances muscle contractions [102]. Moreover, IL-33 in cooperation with IL-13 amplifies muscle responses to ACh [103]. Nevertheless, during helminth infections, these responses to the reflexes need to be restrained to prevent diarrhea and dehydration [103]. This balance is maintained by a combination of ACh degradation through the actions of the hydrolyzing enzyme acetylcholinesterase and through effector mechanisms of the Th_2_ response which leads to decreased serotonin—ACh induced epithelial secretion, as observed during *H. polygyrus*, *N. brasiliensis*, and *T. spiralis* infections [103]. This shows the elegant cooperation of the nervous and immune systems which when disrupted leads to abrogation of the Th_1_/Th_2_ balance and impaired worm expulsion [64] and tissue damage. Serotonin may have one more intriguing role during helminth infection. As it is not a mammal-specific neuromediator and may also be utilized by the tapeworm *Hymenolepis diminuta* [104], it has led to speculation that it may be beneficial for the parasite as a “free” neuromediator [5]. Alternatively, its role in parasite expulsion is evident, therefore its precise role during infection requires further exploration.

Another significant epithelium-derived hormone is cholecystokinin (CCK). It is released by EEC I cells during infection [105] inducing hypophagia, which allows the host to focus on an immune response rather than on digestion. CCK is also an enhancer of gut motility [106]. Stimulation of TLR-2, TLR-4, and TLR-9 [107] as well as taste receptors T1R1—T1R3 [108], likely caused by increased contact of bacteria and amino acids with EEC, induce CCK release. Consequently, CCK may be involved in parasite expulsion via interaction with the microbiota as parasites damage mucus and allow increased microbial access to the epithelium.

## 10. Conclusions

IECs play a significant role in raising, maintaining, and regulating the immune response. They are influential in the response against GI helminths and are an important component of the complicated interplay of immune, neural and hormone systems. Dysregulation of the interactions between these systems can result in immunopathology or immunosuppression.

IECs, with physical contact with parasites, are crucial during the early stages of immune response induction, especially through the release of alarmins (IL-25, IL-33, and TSLP) which prime ILC2 to induce Th_2_ cells and AAM. However, fully defining the role IECs have in inducing the response against parasites requires the identification of the helminth PAMPs that are recognized by IECs and DCs that probe the gut lumen. These remain elusive, and it is possible that separate receptors for helminth PAMPs may not exist. GI helminths interact with TLR2 and TLR4 which are thought to be classical receptors for bacteria, supporting the hypothesis that helminths are not recognized by particular TLR or NLR, but rather a number of signals raised through tissue damage, gut microbiota, and helminth antigens activate appropriate signaling pathways which lead to the “weep and sweep” response.

During the “weep and sweep” response, IECs release mucus which acts as a physical barrier to intruders, detaches parasites from the intestine through an epithelial escalator, increases water influx to the gut lumen, and induce muscle contraction. All these mechanisms are tightly associated with immune and hormone mediators that contribute to maintaining an appropriate Th_2_/Th_reg_ and Th_1_ balance. This balance results in a response strong enough to eradicate intruders and gentle enough to prevent excessive host tissue damage.

Summing up, IECs (among other components—immune cells, muscle, hormone-releasing cells) should be considered as an important node in the complicated interactions that constitute an effective response to GI helminths.

## Data Availability

No new data were collected or analyzed in this study.
